# Intimate Diagnosis of Lessons Affecting the Teeth

**Published:** 1890-12

**Authors:** F. W. Sage

**Affiliations:** Cincinnati, O.


### Intimate Diagnosis of Lesions Affecting the Teeth.

BY F. W. SAGE, D.D.S., CINCINNATI, O.

Read before the Ohio State Dental Society, held at Columbus, October, 1890.

Perhaps in no single respect is the difference betweeen the theory and the practice of medical science so strikingly set forth as by the perplexities which oftentimes beset the practitioner when he undertakes a diagnosis. The questions which at the outset confront him: What is the matter? Where is the exact seat of the trouble? What are the complications? loom up formidably, overshadowing the lesser considerations of the means to be employed for relief. To such a degree is the faculty of insight into the cause and nature of disease superior to every and all other faculties requisite to the successful practice of medicine, that it may be termed the genius of medical instinct towering above the cultured attainments of mere talent. It is the some thing wittily suggested as a prerequisite, in the familiar recipe for dressing a hare, "first catch your hare," the wit of this obviously superfluous admonition inhering in the acknowledged difficulty of accomplishing that feat, and being supplemented by the intimation that it is one matter to know how to do a thing, and another matter, still more important, to supply all requisite conditions for doing it.

The science of diagnosis unlike the science of therapeutics or of prophylactics, can not be reduced to the plane of exactness, because it involves so much that is intangible, indefinable, and irrelevant, that we never really know when we have reached the extreme limits of inquiry. We may, in the midst of our cautious gropings, arrive at data which justify us in excluding from all further consideration this, that, and the other thing, which at first we surmised might be involved in the apparent complications and yet be far from a solution of the problem we are seeking to resolve. Aside from the question of probable causes, and behind and beyond the question of idiosyncracies, lies a vast, unexplored field of contingent causes into which field we may be called to enter, with no guides for our uncertain footsteps other than those which are afforded by the elementary charts which speculation supplies. Nay, more, we may even here be led into error and pursue misleading paths through innocent mirsepresentations of those whose relief we have most at heart; and so, through following false guides, we find ourselves hopelessly involved without being able to determine the point at which the digression was made. What wander is it then that with all the resources of therapeutical science at his command the physician often fails? What wonder is it, that in the midst of variations of symptoms occurring from time to time, no two of half a dozen practitioners are found exactly to agree in their opinions as to what ails the patient? What wonder is it, finally, that the peculiar instinct which by some is esteemed a divine gift—the faculty of quickly determining what is the cause, nature, and location of the disease—should be regarded as marking its possessor as pre-eminent among his colleagues?

The science of diagnosis in its application to the dental branch of medical science is only less important than diagnosis in general in the consequences involved in its right or wrong application. The issues of life and death are perhaps seldom involved in the cases which come under the dentist's attention, and failure to understand the nature of the case or to relieve the symptoms, after all brings to the mortified dentist no apprehension of resultant distresses such as nature can not ultimately relieve.

And that is equivalent to saying that the dentist, like the physician, must often content himself with a tentative course of treatment. He may feel reasonably well assured that a capped pulp is the cause of neuralgic facial pain, and yet through failure to get a response to approved tests, he may consider it expedient to await later developments. This brings us, first of all, to consider a class of difficulties which confront dentist and physician alike; difficulties arising from ignorance, or insufficient knowledge on his part, of what has been done by another who may have had the case in charge. Were time and opportunity always at his command, the dentist who fails to find a way might make it. The patient objects to the experimental removal of a large filling, preferring to take the chances of the dentist being wrong in his speculations. Or he thinks he can better endure present pain than the possible infliction of worse. Upon him then rests the responsibility which the dentist is often quite willing to escape.

The matter of diagnosis in obscure dental lesions is, first of all, a matter of excluding such symptoms as manifestly have no relation to the case. The statements of the patient may or may not aid us; quite as often as otherwise they possess no significance whatever unless they be drawn out from him by leading questions. To get a correct history of the case is not always a simple matter; the patient through fear of an operation which he foresees, or imagines he foresees, suppresses some facts of importance, or perhaps conscious of some negligent or imprudent act of his own, which being confessed would subject him to censure, withholds statements of importance; or again, through feeling of resentment toward yourself, or some other dentist whom he judges blameworthy on account of his present sufferings, even unconsciously exaggerates his symptoms, rather enjoying your perplexity, if he may but succeed in impressing upon you a due sense of your remissness, and a due appreciation of his suffering. What, then, with the querulousness of age, the officiousness of youth, and the innocent ignorance of childhood and infancy, the dentist is liable to be misled in his first approaches to a diagnosis.

But having escaped from, or successfully penetrated all these snares, the next question is, what really are the symptoms? Our inquiries thus far have been made with an intelligent regard for the ignorance, prejudices, and foibles of our patient, so that we know approximately how much to believe, and how much to set aside of his statements. First of all, in every instance where the object is to discover the source of pain, we have learned to be mindful of that most common and ever-to-be-expected phenomenon, reflex nervous irritation. Ask the patient to indicate the aching tooth, and three times out of five he will direct you wrong. Here, then, we must set our general knowledge, acquired by observation and experience, against his most earnest asseverations, and especially after the failure of the usual tests, must reject his opinion in favor of the indications of precedents drawn from our own more extended knowledge of similar cases.

Intimate diagnosis presupposes care and self-watchfulness that we be not unconsciously led into error through our preconceptions. A patient comes in complaining of facial neuralgia. Instantly the dentist's thoughts revert to some occasion, weeks or months before, when with misgivings he capped a pulp for this complaint. Before inviting him to a seat in his chair he has already decided to remove that capping. Such instances of prejudgment based on nothing more than tangible fears of an unfavorable outcome, are by no means rare. Perhaps the dentist invites confirmation of his own false assumption by some remark which gives the patient to understand that he had rather expected trouble with a particular tooth, and is in consequence doubly surprised when he discovers his error. Most important is it then, in all cases, that the dentist admonish himself, first of all, to beware of plausible anticipations, to guard himself against the inferences of mere probability, while at the same time fully recognizing their value so far and *only* so far as they suggest means for intelligent inquiry. He must ever be on the alert for exceptions to the usual rule, for those occasional coincidences which mislead all but the most wide-awake investigators. Perhaps my meaning will be more clearly set forth by a citation of instances in my own office practice.

Miss B, accompanied by her physician, presented herself at my office suffering from anchylosis of the jaw, the result, as it appeared, of an impacted wisdom tooth. A fistula from which pus exuded, under the inferior maxilla, was attributed, by her medical advisor, to necrosis. It was at the patient's solicitation that I was consulted, as I had filled several teeth for her about four years before. Dr.———, the physician having the case in charge had treated her ineffectually for about two months hoping to close the fistula by the use of injections. The anchylosis coming on gradually had reduced the case to a condition so serious as to demand immediate relief, and as the first thing indicated was the extraction of the wisdom tooth, we proceeded with the aid of pine wedges to gain space for that purpose, closure of the jaws to within about half an inch having already taken place. Meanwhile it had occurred to the writer as highly improbable that the fistula proceeded from necrosis at all, or indeed, that it had any connection at all with the wisdom tooth, the roots of which, if not actually curved backwards towards the ramus of the jaw, must have been directed backwards as indicated by the position of the tooth lying with its antero-mesial surface towards the distal surface of the second molar. Several considerations were against the hypothesis of necrosis: first, the single fistula, where necrosis usually exhibits several; second, the probability that the fistula—granting the possibility of its having resulted from alveolar necrosis—would have presented itself on the gum near the tooth, or even have burrowed between the fasciæ and found a more convenient outlet at some point on the neck, or even upon the shoulder or arm; third, the density of the bone in the immediate vicinity of the den sapientia precluding the idea of the pus having burrowed downward and forward to find an outlet directly under the second molar. Seeking confirmation of a suspicion that the second molar was directly implicated, a probe was passed upward from the outside far enough to determine approximately that communication might be made with an abscess associated with the posterior root of the second molar. Following this clew tests were made by tapping, applying heat and cold, etc., to the suspected tooth without the usual response expected, however. The patient declared that hot gutta-percha applied to a gold filling in the crown of this tooth hurt her, at the same time professing no unpleasant or painful sensations when we tapped upon the tooth with a heavy steel instrument. In view of indications already detailed, we chose to ignore these protestations, taking account also of the patient's being extremely nervous and anxious at the same time. The next step was to remove the filling in this second molar tooth, which was found to occupy a fissure cavity hardly a line in depth. At this stage we might have desisted from further search for the dead pulp, there being nothing to suggest its possible exposure at the time of inserting the filling. Determined, however, to pursue the investigation to the very extreme, a very fine probe was employed which was presently made to pass into a minute opening from which pus at once exuded, and we found our suspicions confirmed. Having extracted the tooth we discovered one of those exceptional cases of "elongated" *cornu*, as Garretson terms it, the horns of the pulp being nearly exposed at two other points barely below the enamel line. The patient recovered without further serious inconvenience.

This case illustrates the importance of avoiding such complications as may possibly arise from knowing only half the truth in the outset. Had the wisdom tooth been extracted in this instance, presuming, as is probable, that the anchylosis would have been relieved, the very success of the operation might have entered in as an element to obscure later unfavorable features of the case, so that there is no predicting how much burrowing and chipping away of bone in the suspected socket might have been undertaken to remove necrosed bone where none existed; how much probing and cauterizing of a fistula that had no relation whatever to the wisdom tooth, or the anchylosis might have been employed to torture the despairing victim. To be sure of all these contingent sequelæ would have been in fact avoided in this instance through an accident—the accident of our having decided at all events to extract the second molar. Through that accident might have been revealed the superfluousness of extracting the wisdom tooth also, had no sufficient explanation of the existence of the fistula been before discovered. But through the actual discovery the patient was spared that infliction.

Another element coming in occasionally to frustrate our efforts at a complete diagnosis is born of coincidences. One instance will suffice to illustrate our meaning. A patient applied to the writer recently complaining of an abscessed tooth, the gum and all of the adjacent parts being involved in severe inflammation. He stated that he had consulted another dentist three days before who had opened into the root of the affected tooth—an upper second bicuspid—and once at the time, and again the following day had cleaned and disinfected the root with special care, affording him relief, as he at the time thought, although he admitted when we saw him that he had passed his third night of agony. He said that his dentist had confidently predicted absolute relief, his surprise and chagrin being equaled only by the patient's disappointment on the failure of that prediction. Careful examination disclosed the fact that the treatment of his dentist had been after the usual approved manner, the dressing in the root being free from pus, sweet and dry, so that to all appearances the root was ready for filling. No soreness appeared upon percussion. Suspecting some complication the same test—percussion was tried upon other teeth situated within the field of the swelling and inflammation, revealing not one only, but two other teeth with dead pulps—the canine and the lateral incisor. These having been drilled into pus welled out copiously, giving almost immediate relief. A cure followed their treatment in a very short time.

The idea of excluding certain teeth, suspected through various improvised devices, in order to narrow the field of disturbance and afford greater certainty in forming our diagnosis has sometimes been advantageously employed by the writer. A lady presented herself complaining of pain in a lower bicuspid or molar, which, she was unable to decide. The former had suffered from erosion to the extent of having the enamel largely wasted away, while the gum had receded considerably. The molar had a large cavity in the distal surface, decay having been arrested by a process of eburnation, so that it was not at once practicable to determine whether or not the pulp was alive. Over the bicuspid a piece of French tubing was sprung, the crown being then covered with gutta-percha, so that the tooth was probably perfectly protected from moisture, and from thermal impressions. The cavity in the molar was filled with gutta-percha. In this condition both remained for about a week, no further pain resulting. Finally the bicuspid was covered with a ferrule and cap, and the cavity in the molar permanently filled, both operations being successful. The conclusion at which the writer arrived as the result of this experiment, was that the bicuspid was the offending organ, since had it been the molar, filling even with so excellent a non-conductor as gutta-percha would hardly have availed had the pulp actually been even slightly inflamed. Be that as it may, had the disturbance continued after our having filled the molar, and presuming that nothing had been done to the bicuspid, we might have been still as far as ever from knowing where the trouble lay. The alternative in that event would have been, probably to destroy the molar's pulp, and the sequel to that might still have been the certain discovery that the bicuspid was the source of the offense, showing that the destruction of the pulp in the molar was superfluous. By this simple device we saved our patient pain and ourselves the reproach of empirical practice.

The importance of guarding against uncertain conclusions in the midst of distractions is often illustrated by the discovery that through disregard of reflex disturbances we have located pain in an unoffending organ. Teeth, the roots of which have been filled years before, are often indicated by the patient as the seat of pain which the sequal finally shows proceeds from a remote source; often, indeed, from the inflamed pulp of a tooth in the opposite jaw. Here again, through inability to determine whether or not a root contains a filling—if the patient has been in other hands—the dentist needs to be carefully guarded in forming an opinion. The earnest protestations of the patient will need to be disregarded if the tooth indicated fails to respond to approved tests. I have had patients insist that they plainly felt a swelling at the apex of such teeth where none was noticeable, and have found repeatedly that such complaints ceased, perhaps after treatment of an exposed pulp in a tooth in the opposite jaw. By what perverse freak it is that pain so commonly leaves an offending organ and locates itself in another, once diseased, we can not tell; we know only the general principle that a part having once been the seat of disease is liable to be at times affected falsely in just this way.

My own practice during the past winter (1889) has been unusually prolific in what is popularly termed cold in the teeth, a term properly enough applied as signifying a disposition in teeth known to be sound to ache more or less severely after exposure to cold. I have also noticed a far more than usual number of cases of neuralgia, of a character that subjects the teeth to suspicion, when in reality they are only incidentally implicated, the affection being of a general character requiring the attendance of the physician rather than the dentist.

The length of this paper suggests the propriety of leaving for another occasion the consideration of more serious lesions than such as we have named, and which come under the dentist's observation as presenting phases of at least equal interest.

